# Pathophysiology, Therapeutic Targets, and Future Therapeutic Alternatives in COPD: Focus on the Importance of the Cholinergic System

**DOI:** 10.3390/biom13030476

**Published:** 2023-03-05

**Authors:** Felisbela Gomes, Shih-Lung Cheng

**Affiliations:** 1 Department of Internal Medicine, Far Eastern Memorial Hospital, New Taipei City 22000, Taiwan; 2 Department of Chemical Engineering and Materials Science, Yuan-Ze University, Taoyuan 32056, Taiwan

**Keywords:** chronic obstructive pulmonary disease, inflammation, acetylcholine, muscarinic antagonists, β2 agonists, PDE4 inhibitors

## Abstract

Chronic obstructive pulmonary disease (COPD) is a progressive disease characterized by airway limitation and changes in airway structure. It has a high global burden of mortality and morbidity. The etiology of COPD is complex, but exposure to tobacco smoke and other inhaled lung oxidants are major risk factors. Both pharmacological and non-pharmacological approaches are used to manage COPD, but there remains an urgent unmet need for drugs that can modify the course of the disease. This review focuses on the role of acetylcholine and other components of the pulmonary cholinergic system in the pathogenesis of COPD, and the inhaled pharmacological agents that target it. In addition to its role as a neurotransmitter, acetylcholine regulates diverse aspects of COPD pathogenesis including bronchoconstriction, airway remodeling, mucus secretion and inflammation. Inhaled antimuscarinic drugs are a key component of therapy for COPD, as monotherapy or in combination with inhaled β2 agonists or corticosteroids. We review the evidence supporting the use of current anticholinergic agents in COPD and preview novel drugs targeting the cholinergic system and agents from other classes in clinical development, such as phosphodiesterase-4 inhibitors and monoclonal antibodies targeting inflammatory mediators.

## 1. Introduction

Chronic obstructive pulmonary disease (COPD) is a heterogenous, progressive respiratory disease characterized by airway limitation and changes in airway structures, and/or alveolar destruction (emphysema) [1]. Changes present in the lungs of COPD patients vary, but they may include emphysema (destruction of the alveoli), chronic bronchitis and mucociliary dysfunction [1,2]. Diagnosis of COPD is based on the presence of symptoms such as dyspnea, cough and sputum production, or lower respiratory tract infections lasting longer than expected (≥2 weeks) [2], and the Global Initiative for Chronic Obstructive Lung Disease (GOLD) formally defines COPD as the presence of non-fully reversible airflow limitation forced expiratory volume in one second [FEV_1_]/forced vital capacity [FVC] < 0.7 post-bronchodilation). Patients with COPD are at risk of acute intensification of respiratory symptoms (‘exacerbations’) arising from respiratory infections or environmental triggers that may lead to hospitalization [1]. Asthma and COPD are distinct diseases that may share common clinical features; asthma is a risk factor for COPD, and COPD may coexist alongside asthma in some patients [1]. Patients with COPD often have other chronic diseases, leading to considerable morbidity and mortality [1].

## 2. Epidemiology of COPD

The estimated global burden of COPD in 2019 included 212.3 million prevalent cases and 3.3 million deaths [3]. In 2020, the Global Burden of Diseases, Injuries and Risk Factors Study estimated COPD to be the fourth most common cause of disability-adjusted life years (DALYs) in people 50–75 years and the third most common in those older than 75 years [4]. North America, South Asia and Australasia have the highest point prevalence of COPD (3558.4, 3298.8 and 3192.8 per 100,000, respectively), and East Asia, South Asia and Western Europe have the highest mortality burden (1,264,949, 528,644 and 151,695 deaths, respectively) [3]. Age-standardized point prevalence, deaths and disability-adjusted life years (DALYs) lost due to COPD have decreased in the last 30 years, but absolute burden is increasing, due to population growth and aging [3].

Tobacco smoking is the strongest risk factor for developing COPD, estimated to be responsible for 46.0% of cases globally [3,5]. However, a substantial number of COPD patients are non- or never-smokers. An international survey of over 4000 subjects found a prevalence of COPD of 12.2% in never-smokers, and never-smokers comprised 27.7% of all COPD cases [6]. Exposure to ambient particulates or occupation exposure to particulates, gases and fumes are also a notable risk factors, responsible for 20.7% and 15.6% of cases, respectively [3,5]. Smoking cessation and drugs available should be necessary to avoid smoking-related lung parenchymal injury. Genetic risk factors have been identified, most notably, severe hereditary deficiency of alpha-1 antitrypsin [7,8].

## 3. Non-Pharmacological Management of COPD

Smoking cessation is considered to be the most effective strategy for preventing or slowing the progression of COPD, although data suggest COPD patients may have more difficulty quitting using standard cessation techniques [9]. Approaches that combine behavioral and pharmacological interventions can be effective and should be included as part of a comprehensive management of the disease [9,10]. Pulmonary rehabilitation exercises can improve exercise capacity in selected patients, and a meta-analysis concluded that various forms can be effective, including urban training (circuits to encourage daily walking), active mind–body movement therapies (e.g., yoga and tai chi), and Pilates [11]. Long-term use of oxygen therapy can increase survival in patients with severe resting hypoxemia [12], and while oxygen can relieve exercise-induced breathlessness in patients with daily activity and enhance the benefits for pulmonary rehabilitation. Surgical interventions, namely, lung volume reduction or lung transplantation, may be warranted in more severely affected COPD patients [2].

## 4. Pharmacological Approaches to Stable COPD

Pharmacological therapy for COPD aims to alleviate symptoms, reduce the frequency and severity of exacerbations and improve health status. A meta-analysis of nine studies (n = 33,051) of at least a 1-year duration using a variety of pharmacological classes, concluded pharmacotherapy can reduce the rate of lung function decline, measured by FEV_1_, versus placebo [13]. Pharmacological classes used to treat COPD include inhaled bronchodilators, particularly antimuscarinic drugs, inhaled or oral corticosteroids, phosphodiesterase-4 (PDE4) inhibitors and mucolytic agents. Treatment is individualized based on the patient’s severity of disease symptoms and history of exacerbations; the selection of treatment options is guided by cost, availability and favorable response versus side effects [1]. To guide initial therapy, the GOLD 2023 guidelines categorize patients into Groups A, B and E based on their history and severity of exacerbations, and dyspnea severity is measured by the modified Medical Research Council (mMRC) dyspnea questionnaire or the COPD Assessment Test (CAT) [1]. Current options for the management of COPD reduce symptoms, but there is an urgent unmet need for treatments that can modify the underlying disease processes [2].

## 5. The Pulmonary Cholinergic System

### 5.1. Components of the Cholinergic System

The role of acetylcholine (ACh) has been well studied in the context of neurotransmission, but it also has a considerable role in non-neuronal processes, and its components are widely expressed non-neuronal tissues [14]. Dysregulation of the cholinergic system is implicated in diseases affecting various organ systems including respiratory diseases [14]. In the lungs, ACh is produced by both neuronal and nonneuronal cells, such as endothelial cells, keratinocytes and lymphocytes [15]. A distinction between the function of neuronal and non-neuronal ACh is extremely difficult, and the extracellular concentration of ACh released by epithelial cells is expected to be several orders of magnitude lower than that surrounding stimulated nerve fibers [16]. Choline acetyltransferase (ChAT), the enzyme that synthesizes ACh from choline and acetyl-coenzyme-A, is expressed in diverse forms encoded by the same gene [16]; in the lungs, specific forms of ChAT are expressed in neurons, and reverse transcription polymerase chain reaction (RT-PCR) and immunohistochemistry data suggest a single variant of ChAT is expressed in various airway epithelial cell types [16]. In neurotransmission, the activity of ACh is terminated by hydrolysis by acetylcholinesterase (AChE) or butyrylcholinesterase (BChE). In the lungs, their expression appears to be limited to neurons, and ACh degradation is largely absent in airway epithelia [16]. Vesicular acetylcholine transporter (VAChT)—a transmembrane protein mediating storage of ACh at synapses—is an important component of ACh recycling at synapses, but it is not typically found in non-neuronal cells [16].

The actions of ACh in neuronal and non-neuronal cells are mediated by G-protein coupled transmembrane receptors, known as muscarinic acetylcholine receptors (mAChR) and ligand-gated ion channels classified as nicotinic acetylcholine receptors (nAChR) [15]. In the lungs, five subtypes of mAChR are expressed (M_1_–M_5_), but only M_1_, M_2_ and M_3_ have a well-understood function; muscarinic receptors are expressed in parasympathetic nerves, mucus glands and smooth muscle cells [15]. Almost all known nAChR subunits have been detected in tissues from the mammalian respiratory tract, including epithelial, muscle, nerve and connective tissues [17,18]. Although nicotinic receptors are important in neurotransmission in the lungs, muscarinic receptors are the primary target for therapies for COPD and other airway diseases [15].

### 5.2. Non-Neuronal Functions of the Pulmonary Cholinergic System

In addition to its classical role in modulating neurotransmission across preganglionic/postganglionic synapses, ACh has diverse roles in the tissues of the lungs [19]. In the pulmonary arteries, ACh stimulation increases intracellular calcium, and induces vasodilation, likely via the stimulation of nitric oxide production [15]. In the airway smooth muscles, ACh from parasympathetic nerves acts via the M_3_ receptor to cause smooth muscle contraction leading to bronchoconstriction, and stimulation of the M_2_ receptor also contributes to bronchoconstriction by inhibiting smooth muscle relaxation [15]. Activation of muscarinic receptors in the airway epithelial cells increases ciliary beat frequency, enhancing transport of mucus and particulates from the lung [20], and activation of M_3_ muscarinic receptors in tracheal submucosal glands induces a rapid increase in mucus secretion [21].

### 5.3. The Pathophysiological Role of the Cholinergic System in COPD

**Figure 1:**
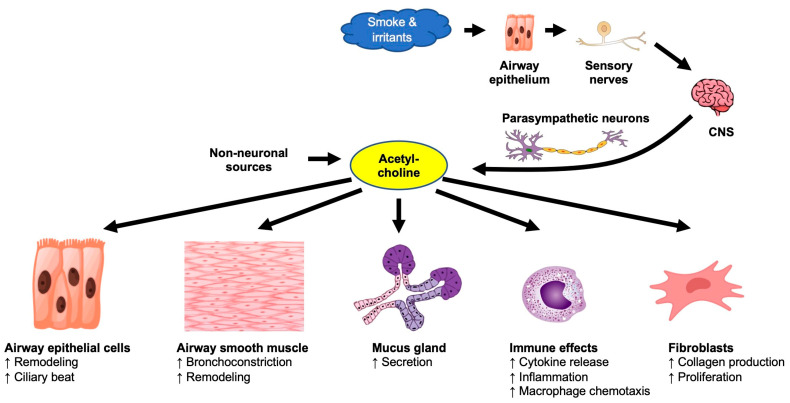
Acetylcholine in the pathogenesis of COPD. Adapted from Buels et al., 2012 [15], Kummer et al., 2008 [16], and Kolahian et al., 2012 [22].

The etiology of COPD is very complex and heterogenous; data from model systems suggest the pulmonary cholinergic system contribute widely to underlying processes of these diseases, including inflammation, bronchoconstriction, mucus production and remodeling of airways (Figure 1) [15,16,22]. Initially, lung irritants (e.g., smoke, cold air and dust) trigger signals, sent via afferent sensory nerves and the vagus nerve, to the brain, leading to parasympathetic nerves conducting a reflex response back to the lungs [15]. This reflex response drives ACh release by airway neurons that acts via M_3_ receptors to promote excessive bronchoconstriction and mucus secretion [15].

The cholinergic system also contributes to the inflammatory response and tissue remodeling seen in COPD patients, with components of the cholinergic system implicated in the activation of both inflammatory and anti-inflammatory responses. Stimulation of the M_3_ receptor in cultured alveolar macrophages results in proinflammatory chemotactic activity [23], whereas stimulation of nicotinic receptors suppresses proinflammatory activity of alveolar macrophages [24]. In a small clinical trial, targeted radiofrequency ablation of lung nerves reduced inflammation in patients with moderate-to-severe COPD, suggesting a key role for neuronal ACh in lung inflammation [25]. 

The pulmonary cholinergic system also contributes to the structural changes seen in airways of patients with COPD. In cultured fibroblast cells, stimulation of M_2_ and M_3_ receptors induces collagen production and cell proliferation by fibroblasts [26,27,28]. Cell culture models also suggest ACh can enhance proliferation of smooth muscle induced by other growth factors [29]. In addition, acetylcholine is the primary parasympathetic neurotransmitter in the airways, where it not only induces bronchoconstriction and mucus secretion, but it also regulates airway epithelial cells and remodeling. Acetylcholine promotes inflammation and remodeling via direct effects on airway epithelial cells, and via mechanical stress applied to the airways sequential to bronchoconstriction. The effects on inflammation and remodeling are regulated by both neuronal and non-neuronal acetylcholine mechanism. Taken together, we demonstrated that the combined effects of anticholinergic therapy on M3-mediated bronchoconstriction, mucus secretion, inflammation and remodeling may enhance the treatment responses with these drugs for patients with chronic pulmonary obstructive disease (COPD).

### 5.4. Mucus Plugs and Small Airway Dysfunction in COPD

Recent studies have demonstrated that mucus plugs and small airway dysfunction are essential pathogenesis for COPD [30,31]. These abnormalities may be associated with cho-linergic system related airway inflammation and airway remodeling. 

## 6. Anticholinergic Medications in the Treatment of COPD

In healthy airways, the different subtypes of muscarinic receptors, may either supplement or counteract each other’s actions. For example, the M_2_ receptors on postganglionic parasympathetic nerves inhibit ACh release, but this activity is counteracted by M_1_ receptors in ganglia, which increase ACh release by facilitating neurotransmission [15]. An example of supplementary action of different mAChR subtypes is seen in the smooth muscle of the airways, where the activity of the M_2_ receptor supplements contraction caused by activation of the M_3_ receptor [15]. This suggests a model where in healthy airway tissues, balanced activity of the various mAChR subtypes achieves and maintains appropriate muscle tone, mucus secretion and mucociliary clearance, and disruption of this balance may lead to COPD. 

Although preclinical studies have shown strategies targeting nicotinic receptors, particularly α_7_, to be promising, this approach has not yet been translated into clinical trials [18]. In obstructive airway disease, the role of muscarinic acetylcholine receptors, particularly M_3_, in excessive bronchoconstriction and mucus secretion make them a logical therapeutic target [15]. Muscarinic antagonists competitively bind muscarinic receptors, blocking the action of ACh [15,32]. Inhaled formulations of muscarinic antagonists are classified into short-acting muscarinic antagonists (SAMAs; 6–9 h duration) such as ipratropium bromide and oxitropium bromide, and long-acting muscarinic antagonists (LAMAs; 12–24 h duration) including aclidinium bromide, glycopyrronium bromide, tiotropium, and umeclidinium [1]. Inhaled SAMAs and LABAs are a mainstay of therapy for patients with COPD, and they may be used in combination with other inhalable agents such as short- or long-acting β2 agonists (SABA/LABA), or inhaled corticosteroids (ICS).

## 7. Management of COPD Patients with Inhaled Bronchodilators 

**Figure 2:**
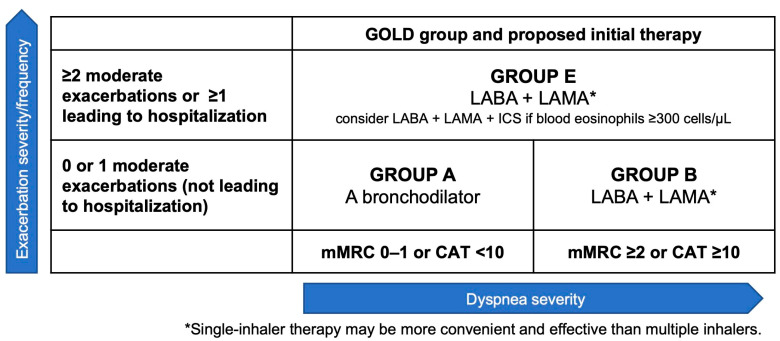
Recommendations for initial bronchodilator therapy from the 2023 Global Initiative for Chronic Obstructive Lung Disease. Adapted from GOLD 2023 [1]. CAT, COPD Assessment Test; GOLD, Global Initiative for Chronic Obstructive Lung Disease; LABA, long-acting β2 agonist; LAMA, long-acting muscarinic antagonist; mMRC, modified Medical Research Council dyspnea questionnaire.

The 2023 GOLD Report proposes that the initial therapy choice in COPD is guided by the patient’s frequency and severity of exacerbations, and severity of dyspnea (Figure 2) to classify into Group A, Group B and Group E. [1]. Group A patients—those with 0–1 exacerbations not needing hospitalization and milder dyspnea—should initially be treated with a single bronchodilator. Group B patients are defined by the same exacerbation history as group A, but with more severe dyspnea, and they should initially receive a LABA plus LAMA combination therapy. Patients who have experienced two or more exacerbations (of any severity) or one exacerbation or more requiring hospitalization should receive a LABA plus LAMA as the initial therapy irrespective of dyspnea status, with an initial LABA plus LAMA plus ICS (‘triple therapy’) to be considered for those with blood eosinophils ≥ 300 cells/μL. For inhaled therapies to be effective, it is crucial that patients receive appropriate education on how to use their inhaler and the importance of correct technique, and the patient’s inhaler technique should be checked before concluding their current therapy is inadequate. A range of inhaled formulations of bronchodilatory drugs targeting the pulmonary cholinergic system, as monotherapy or in combination with other drug classes, are available for treatment of COPD, including single-, dual- or triple-agent combinations (Table 1) [1].

The use of SAMAs and LAMAs for treatment of COPD is supported by extensive clinical evidence. For example, the efficacy of ipratropium bromide is supported by a meta-analysis of seven studies comparing it to LABAs that concluded both agents had a similar effect on alleviating COPD symptoms [33]. In a meta-analysis of 22 studies, tiotropium demonstrated improved quality of life, as measured by the St George’s Respiratory Questionnaire (scale 0–100; mean difference, −2.89; 95% confidence interval [CI], −3.35 to −2.44), reduced the number of patients with exacerbations (odds ratio [OR], 0.78; 95% CI, 0.70 to 0.87), and reduced the risk of hospitalizations due to exacerbations compared to placebo (OR, 0.85; 95% CI, 0.72 to 1.00) [34]. A meta-analysis of two studies showed that tiotropium treatment was associated with improved lung function (mean FEV_1_ difference of 109 mL), fewer hospital admissions (OR, 0.34; 95% CI, 0.15 to 0.70), and fewer exacerbations leading to hospitalization (OR, 0.56; 95% CI, 0.31 to 0.99) compared to ipratropium [35]. This meta-analysis also showed there were fewer non-fatal serious adverse events (AEs) in the tiotropium group compared with the ipratropium group (OR, 0.5; 95% CI, 0.34 to 0.73) [35]. Clinical trials also suggest tiotropium has a greater effect on exacerbation rates than LABAs (salmeterol, indacaterol) [36,37]. 

Evidence of the efficacy of aclidinium in COPD is exemplified by a meta-analysis of 21 studies that concluded that aclidinium twice daily produced similar improvements in lung function, dyspnea and quality of life to tiotropium once daily or glycopyrronium once daily [38].

Revefenacin is a nebulized once-daily LAMA that acts via the M_3_ receptor [39]; its use for COPD was approved by the US Food and Drug Administration (FDA) for COPD in 2018 [40]. Revefenacin has been evaluated in phase II/III placebo-controlled trials, and active-comparator trials against tiotropium [40]. Revefenacin produced statistically significant improvements in lung function versus placebo and had similar AE rates to tiotropium [40]. A meta-analysis of nine randomized studies (both placebo and active comparator) concluded that although revefenacin did offer benefits over placebo, better quality evidence was needed to compare its efficacy to that of tiotropium [41].

### 7.1. Combination of Anticholinergic Drugs with β2 Agonists in COPD

If patients are unable to achieve symptom control on monotherapy, a common dual-therapy approach is combining anticholinergic agents with β2 agonists of similar duration (i.e., SABA + SAMA, or LABA + LAMA). Upon binding to β2 adrenergic receptors, SABAs and LABAs initiate a signal cascade that increases intracellular cyclic adenosine monophosphate (cAMP), ultimately leading to prevention of smooth muscle contraction and bronchodilation [42]. In a 6-week randomized cross-over study, combined albuterol plus ipratropium was superior to either alone for improving FEV_1_ (24% and 37% more vs. albuterol or ipratropium monotherapy, respectively; both *p* < 0.001), while maintaining a similar AE profile [43]. A meta-analysis of 24 studies concluded LABA plus LAMA combination therapy was superior to either class as monotherapy for reduction in exacerbations (20% reduction; *p* < 0.002) and hospitalizations (11% reduction; *p* < 0.01); no differences were observed for mortality, AEs or pneumonia [44]. Beneficial effects of tiotropium plus olodaterol on lung rehabilitation have been reported in a meta-/pooled analysis of a large clinical trial program [45]. These benefits included reduced lung hyperinflation, increased exercise endurance capacity and improved patient perception of dyspnea [45].

### 7.2. The Addition of Inhaled Corticosteroids to Single-Bronchodilator Therapy

Corticosteroids act through multiple mechanisms, including changes in gene expression and cell recruitment to suppress inflammatory processes in the lungs [46]. The 2023 GOLD report supports the consideration of ICS for patients with a history of COPD hospitalization, ≥2 moderate exacerbations annually, blood eosinophils ≥ 300 cells/μL, or history of concomitant asthma [1]. Although ICS alone is not an effective treatment for COPD [47], the addition of ICS to LABA is more effective versus placebo or LABA monotherapy for the reduction in exacerbations, improvement of lung function and quality of life [48,49,50,51]. A meta-analysis of nine studies comparing single-inhaler LABA plus ICS to LABA alone concluded there was low-quality evidence of reduced exacerbation rates with LABA plus ICS (rate ratio [RR], 0.76; 95% CI, 0.68 to 0.84) and moderate-quality evidence of increased quality of life [52].

A meta-analysis including six studies comparing LABA plus LAMA with LAMA monotherapy or LABA plus ICS concluded LABA plus LAMA significantly improved FEV_1_ and exacerbation rates versus LAMA or LABA plus ICS (0.07 L and 0.08 L, respectively; *p* < 0.0001) [53]. The AE rate was no different for LABA plus LAMA versus LAMA alone, but it was lower versus LABA plus ICS (RR, 0.82; 95% CI, 0.75 to 0.91) [53].

Although previous GOLD Reports suggested LABA plus ICS as initial therapy for some patients, this was revised in the 2023 edition, and bronchodilator monotherapy or LABA plus LAMA is now suggested as the initial starting therapy for the majority of patients [1]. Studies evaluating LAMA plus ICS combinations are scarce, and data from a pre-clinical study did not suggest patients would benefit from this combination [54]. However, several studies of tiotropium plus ICS combinations suggest some patients might benefit from the addition of ICS to a LAMA [55].

### 7.3. Triple Therapy for COPD

The addition of ICS to LABA plus LAMA (‘triple therapy’) should be considered in patients with moderate-to-severe disease. Three fixed-dose combinations are widely available: fluticasone plus umeclidinium plus vilanterol, beclomethasone plus formoterol plus glycopyrronium, and budesonide plus formoterol plus glycopyrrolate [1]. These combinations have been validated by several large, long-term randomized studies comparing them to dual therapies in moderate-to-severe COPD. The IMPACT study compared fluticasone plus umeclidinium plus vilanterol once daily to fluticasone plus vilanterol, or umeclidinium plus vilanterol [56]. Triple therapy resulted in lower rates of moderate or severe COPD exacerbation than fluticasone plus vilanterol (RR, 0.85; 95% CI 0.80 to 0.90) or umeclidinium plus vilanterol (0.75; 95% CI, 0.70 to 0.81) [56]. In the TRIBUTE study, triple therapy with beclomethasone plus formoterol plus glycopyrronium once daily significantly reduced the rate of moderate-to-severe exacerbations compared with indacaterol plus glycopyrronium (RR, 0.848; 95% CI 0.723 to 0.995) [57]. A further example of the efficacy of triple therapy is the ETHOS study, which compared two doses of budesonide (320 or 160 μg) plus formoterol plus glycopyrrolate to two dual therapies (glycopyrrolate + formoterol or budesonide + formoterol) [58]. Triple therapy with either 320 or 160 μg budesonide resulted in a lower rate of moderate or severe exacerbations that was achieved with the dual-therapy regimens [58].

In the TRIBUTE, IMPACT and ETHOS studies, overall AE rates were similar between triple- and dual-therapy regimens [56,57,58], but ETHOS and IMPACT reported a higher rate of pneumonia in patients receiving corticosteroid-containing combinations [56,58]. In IMPACT, the glucocorticoid-containing regimens (fluticasone + umeclidinium + vilanterol and fluticasone + vilanterol) had higher rates of pneumonia than the umeclidinium plus vilanterol group (8%, 7% and 5%, respectively). The risk of clinician-diagnosed pneumonia was higher with triple therapy compared to umeclidinium plus vilanterol (HR, 1.53; 95% CI, 1.22 to 1.92; *p* < 0.001), but no significant difference was reported in the risk of pneumonia between triple therapy and fluticasone plus vilanterol (HR, 1.02; 95% CI, 0.87 to 1.19; *p* = 0.85) [56]. In ETHOS, pneumonia rates were 4.2% and 3.5% in the triple therapy groups (budesonide 320 μg or 160 μg + formoterol + glycopyrrolate, respectively), 2.3% in the glycopyrrolate plus formoterol group and 4.5% in the budesonide plus formoterol group [58]. Time to first pneumonia event was significantly longer in the glycopyrrolate plus formoterol group compared to groups treated with glucocorticoid-containing combinations (*p* < 0.05) [58].

Notably, the IMPACT and ETHOS studies (in the 320 μg budesonide group) demonstrated reductions in mortality, a secondary outcome, with triple therapy versus LAMA plus LABA dual therapy [56,59]. However, in these studies, no mortality difference was seen between triple therapy and LABA plus ICS [56,59]; a post hoc pooled analysis of three studies of triple therapy also reached similar conclusions [60]. While promising, these data should be interpreted with caution due to limitations in study design, particularly the potential effect of discontinuation of current treatment at study enrolment [61]. Future studies designed with mortality as the primary endpoint may resolve this limitation.

## 8. Non-Cholinergic Targets for COPD Treatment

### 8.1. Therapies Targeting Phosphodiesterase-4 (PDE4) in COPD

**Figure 3:**
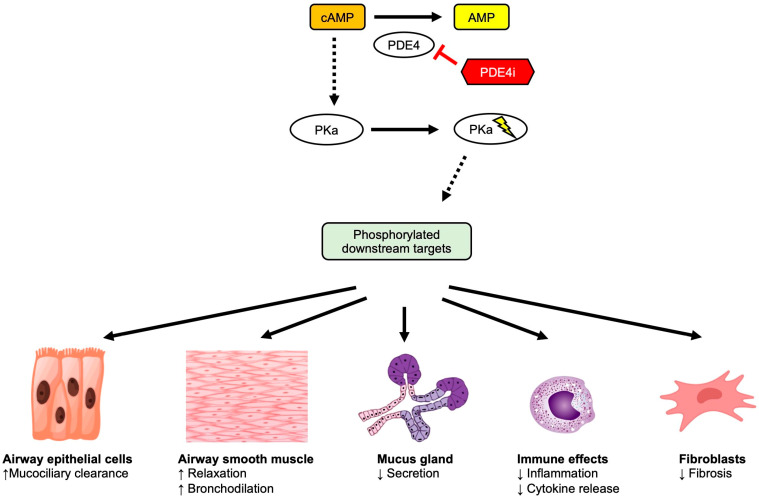
Beneficial effects of PDE4 inhibition in airway diseases. Adapted from Contreras et al., 2017 [62] and Joskova et al., 2020 [63]. (c)AMP, (cyclic) adenosine monophosphate; PDE4(i), phosphodiesterase-4 (inhibitor); PKa, protein kinase A.

The enzyme PDE4 is implicated in the degradation of cAMP in most types of immune and inflammatory cells. PDE4 inhibitors elevate intracellular levels of Camp, activating protein kinase A (Pka) leading to phosphorylation of downstream targets [62]. This leads to diverse effects in respiratory tissues, including inhibition of the proinflammatory immune response by macrophages, T lymphocytes and neutrophils [62]. PDE4 inhibition also has bronchodilatory effects, beneficial effects on fibrosis, and beneficial effects on mucociliary clearance (Figure 3) [62,63]. Although the interaction of PDE4 inhibition on the cholinergic system has mostly been examined in neurons, limited data suggest PDE4 inhibition has effects that counteract those of Ach in the lungs. Airways of mice genetically deficient for *PDE4D* are unresponsive to cholinergic stimulation and do not show airway hyperreactivity to antigens, even though components of the inflammatory response such as antigen sensitization and inflammatory cell infiltration of the lungs appear to be normal [64]. Rolipram, a PDE4 inhibitor, can relax ACh-mediated contraction in tracheal tissue of guinea pigs [65], and quercetin (a naturally occurring PDE4 inhibitor) prevents ACh-mediated contraction of tracheal tissue in mice [66].

In two randomized placebo-controlled studies, the oral PDE4 inhibitor roflumilast (added to SABA, LABA or SAMA background therapy) reduced moderate-to-severe exacerbations by 17% versus placebo in patients with moderate-to-severe COPD [67]. The 2023 GOLD 2023 guidelines suggest that roflumilast (a PDE4i) may be used as an add-on therapy for patients with persistent symptoms/exacerbations despite optimal management (e.g., LABA + LAMA, or triple combinations) [1].

Currently, roflumilast is the only PDE4i approved for treatment of COPD, and its gastrointestinal (GI) AEs limit its use to patients with more severe disease [68,69]. Therefore, development of novel PDE4 inhibitors for COPD centers on strategies to circumvent this problem, including inhaled PDE4 inhibitors and more selective inhibitors that avoid inhibiting PDE4D, an isoform of PDE4 whose inhibition is thought to be the predominant cause of GI effects [70]. Tanimilast (CHF6001) is an inhaled nonselective PDE4i which has passed phase II studies for asthma and COPD, and it is currently undergoing phase III studies as an add-on to triple therapy [71]. Data up to phase II suggest that inhaled tanimilast avoids the GI side effects observed with oral roflumilast [71]. BI1015550 is an oral selective inhibitor of PDE4B and has both anti-inflammatory and anti-fibrotic activity in model systems [72]. A placebo-controlled phase II study in patients with idiopathic pulmonary fibrosis (IPF) demonstrated that BI1015550 prevented decline in lung function compared with placebo while maintaining an acceptable safety profile [73]; phase III studies in IPF are ongoing. Ensifentrine (RPL554) is an inhaled dual PDE3/4 inhibitor that has been evaluated in a phase II dose-ranging study and a phase II study in which ensifentrine was combined with salbutamol, ipratropium, or tiotropium. Ensifentrine produced additional bronchodilatory effects and reduction in hyperinflation when combined with standard bronchodilators [74,75]. Furthermore, limited data have also been announced from two placebo-controlled phase III studies (ENHANCE-1 and ENHANCE-2); ensifentrine met the primary endpoint with a placebo-corrected change in FEV_1_ AUC_0–12 h_ from baseline of 87 mL at Week 12, as well as key secondary endpoints demonstrating improvements in symptoms and quality of life [76].

### 8.2. Development of Novel Bronchodilators for COPD

Muscarinic antagonist/β2 agonists (MABAs) are a new class of bronchodilators in development that combine anticholinergic and β2-agonistic mechanisms of bronchodilation into a single molecule [77]. Batefenterol (GSK961081) is an example that is in clinical development for COPD. A phase II study has demonstrated lung function improvements compared with placebo with an acceptable tolerability profile in patients treated with inhaled batefenterol plus fluticasone once daily [78]. Navafenterol (AZD8871) is a MABA that has undergone a phase IIa three-way crossover study that compared 2 weeks’ treatment with inhaled navafenterol, inhaled umeclidinium plus vilanterol, and placebo [79]. Navafenterol was well tolerated and demonstrated significant improvements in trough FEV_1_ versus placebo (mean difference 0.202 L; *p* < 0.0001); no significant difference versus umeclidinium plus vilanterol was observed [79]. It is currently unclear if clinical development of batefenterol or navafenterol will proceed further. Several other MABAs are in earlier stages of clinical development [80].

### 8.3. Monoclonal Antibodies and Biologics for COPD

From 2003 to 2021, five monoclonal antibodies (mABs) have been approved by the US FDA for severe asthma or pediatric asthma patients not achieving control on inhaled therapies, namely, omalizumab (anti-immunoglobulin E), mepolizumab (anti-interleukin [IL] -5), benralizumab (anti-IL-5 receptor), dupilumab (anti-IL4 receptor) and tezepelumab (anti-thymic stromal lymphopoietin [TSLP]) [81]. Most mABs are currently administered subcutaneously; inhaled formulations have potential advantages of low systemic exposure and matching delivery route to site of action, but effectively delivering mABs via inhalation remains technically challenging and will require the development of specialized formulations [82].

Due to pathological similarities between asthma and COPD, mABs approved for severe asthma are undergoing clinical evaluation for COPD (Table 2). In asthma, inflammation is mainly due to the activity of type 2 T-helper cells, which produce multiple cytokines (notably IL-4, IL-5 and IL-13) with diverse roles in the inflammatory process [83]. Interleukins -4, -5 and -13 all contribute to eosinophil recruitment and can be therapeutically targeted with mABs [83]. The IL-5 pathway can be targeted with mepolizumab, a mAB against free IL-5, and benralizumab, a mAB against its receptor (IL-5R) [84]. Multiple large, randomized studies have evaluated these two agents in COPD; a Cochrane systematic review and meta-analysis of six studies (three each for mepolizumab and benralizumab) concluded both agents are likely to reduce the rate of exacerbations in COPD patients with high eosinophil counts [85]. However, neither agent is currently licensed for COPD, and the optimal method of identifying the subpopulation of COPD patients most likely to benefit from IL-5-directed therapies is unclear [86].

Dupilumab is a mAB inhibiting IL-4R, a component of the type 2 receptor complex (IL-4/IL-13) that regulates recruitment of eosinophils as well as airway remodeling in asthma [89]. Two clinical studies (BOREAS and NOTUS) are evaluating its activity on exacerbation rates in patients with moderate-to-severe COPD and type 2 inflammation [89]. TSLP is an upstream cytokine involved in type 2 inflammation and other pathogenic processes in COPD [90]. Tezepelumab (anti-TSLP) is being evaluated in a phase II placebo-controlled randomized study (COURSE) in COPD patients with exacerbations on triple therapy [84].

In addition to mABs approved for asthma, other biologics targeting inflammatory pathways have undergone evaluation for COPD. Infliximab, an inhibitor of soluble and membrane-bound tumor necrosis factor-α (TNF-α) has been evaluated in phase 2 studies enrolling patients with mild, moderate and severe COPD, but no clinical benefits were reported [91,92]. Furthermore, a randomized phase 2 study concluded that etanercept, a biologic inhibitor of soluble TNF-α, did not show any efficacy benefit over oral prednisone for acute exacerbations of COPD [93]. Interleukin 1β (IL-1β) is strongly implicated in the pathogenesis of COPD and exacerbations [94], but early-phase placebo-controlled studies of canakinumab (anti-IL-1β) or MEDI8968 (a mAB binding to IL-1β) have not shown efficacy in patients with COPD [95]. A placebo-controlled pilot study of ABX-CXCL8, a human anti-IL-8 mAB did suggest a beneficial effect on dyspnea, but no effect on lung function or health status [96]. Two subtypes of IL-17, IL-17A and IL-17C are implicated in the recruitment of inflammatory cells in COPD [97], and a mAB against IL17A (CNTO6785) has been evaluated for COPD in a placebo-controlled phase II study, but no evidence of clinical benefit was found [98].

## 9. Conclusions

In this review, we focus on the role of acetylcholine and other components of the pulmonary cholinergic system in the pathogenesis of COPD, especially in the epithelial cell inflammation, mucus plugs, small airway dysfunction and remodeling. We also emphasize the anti-cholinergic agents for treatment of COPD. Currently, COPD is a disease with a high unmet need for therapies that modify the underlying disease processes rather than alleviate symptoms. As the pathogenesis of COPD is further elucidated, new data will assist the development of novel agents targeting both the cholinergic system and other pathways contributing to the disease. A better understanding of disease biomarkers may inform a targeted approach to matching individual COPD patients to the most appropriate selection of therapy.

The ambiguous clinical efficacy of mABs for COPD in trials thus far is likely due to a redundancy in pathways responsible for inflammation, as well as the heterogenous populations recruited into large clinical trials. Trials enrolling more narrowly defined populations may be of great value in identifying the precise role of biologics in the future treatment strategies for COPD.

## Figures and Tables

**Table 1:** Short- and Long-Acting Bronchodilator Formulations That Contain Muscarinic An-Tagonists. Adapted from GOLD 2023 [1].

Drug Class	Drug(s)	Inhaler Type	Duration of Action (h)
Short-acting muscarinic antagonist (SAMA)	Ipratropium bromide	MDI	6–8
	Oxitropium bromide	MDI	7–9
Long-acting muscarinic antagonist (LAMA)	Aclidinium bromide	DPI, MDI	12
	Glycopyrronium bromide	DPI	12–24
	Tiotropium	DPI, SMI, MDI	24
	Umeclidinium	DPI	24
	Glycopyrrolate	(nebulizer only)	12
	Revefenacin	(nebulizer only)	24
Combination short-acting β_2_ agonist + short-acting muscarinic antagonist (SABA + SAMA)	Fenoterol/ipratropium	SMI	6–8
	Salbutamol/ipratropium	SMI, MDI	6–8
Combination long-acting β_2_ agonist + long-acting muscarinic antagonist (LABA + LAMA)	Formoterol/aclidinium	DPI	12
	Formoterol/glycopyrronium	MDI	12
	Indacaterol/glycopyrronium	DPI	12–24
	Vilanterol/umeclidinium	DPI	24
	Olodaterol/tiotropium	DPI	24
Combination long-acting β_2_ agonist + long-acting muscarinic antagonist + inhaled corticosteroid (Triple therapy)	Fluticasone/umeclidinium/ vilanterol	DPI	24
	Beclometasone/formoterol/ glycopyrronium	MDI	12
	Budesonide/formoterol/ glycopyrrolate	MDI	12

DPI, dry powder inhaler; LABA, long-acting β2 agonist; LAMA, long-acting muscarinic antagonist; MDI, metered dose inhaler; SMI, soft mist inhaler.

**Table 2:** A summary of ongoing trials of assessing mABs approved for asthma in the treatment of COPD.

Antibody	Target	Results in COPD to Date	Ongoing Studies
Mepolizumab	IL-5	Reduced exacerbation rates in patients with high eosinophil counts [87]	MATINEE (NCT04133909) COPD-HELP (NCT04075331)
Benralizumab	IL-5R	Reduced exacerbation rates in patients with high eosinophil counts [88]	RESOLUTE (NCT04053634) ABRA (NCT04098718)
Dupilumab	IL-4		BOREAS (NCT03930732) NOTUS (NCT04456673)
Tezepelumab	TSLP		COURSE (NCT04039113)

IL, interleukin; TSLP, thymic stromal lymphopoietin.

## Data Availability

Not applicable.
